# Bioinspired Dielectric Film with Superior Mechanical Properties and Ultrahigh Electric Breakdown Strength Made from Aramid Nanofibers and Alumina Nanoplates

**DOI:** 10.3390/polym13183093

**Published:** 2021-09-14

**Authors:** Qiu-Wanyu Qing, Cheng-Mei Wei, Qi-Han Li, Rui Liu, Zong-Xi Zhang, Jun-Wen Ren

**Affiliations:** 1 College of Electrical Engineering, Sichuan University, Chengdu 610065, China; 2018141441129@stu.scu.edu.cn (Q.-W.Q.); weichengmei@stu.scu.edu.cn (C.-M.W.); 2 College of Aviation Engineering, Civil Aviation Flight University of China, Guanghan 618307, China; cunzhangfangyang@163.com; 3 State Grid Sichuan Electric Power Research Institute, Chengdu 610072, China; mbchaoren@163.com (R.L.); 2019223035137@stu.scu.edu.cn (Z.-X.Z.)

**Keywords:** aramid nanofiber, hydrogen bonds, electric breakdown strength, mechanical strength, alumina nanoplates

## Abstract

Materials with excellent thermal stability, mechanical, and insulating properties are highly desirable for electrical equipment with high voltage and high power. However, simultaneously integrating these performance portfolios into a single material remains a great challenge. Here, we describe a new strategy to prepare composite film by combining one-dimensional (1D) rigid aramid nanofiber (ANF) with 2D alumina (Al_2_O_3_) nanoplates using the carboxylated chitosan acting as hydrogen bonding donors as well as soft interlocking agent. A biomimetic nacreous ‘brick-and-mortar’ structure with a 3D hydrogen bonding network is constructed in the obtained ANF/chitosan/Al_2_O_3_ composite films, which provides the composite films with exceptional mechanical and dielectric properties. The ANF/chitosan/Al_2_O_3_ composite film exhibits an ultrahigh electric breakdown strength of 320.1 kV/mm at 15 wt % Al_2_O_3_ loading, which is 50.6% higher than that of the neat ANF film. Meanwhile, a large elongation at break of 17.22% is achieved for the composite film, integrated with high tensile strength (~233 MPa), low dielectric loss (<0.02), and remarkable thermal stability. These findings shed new light on the fabrication of multifunctional insulating materials and broaden their practical applications in the field of advanced electrics and electrical devices.

## 1. Introduction

Polymers-based dielectrics are widely utilized in advanced electronics and electric power systems by virtue of their irreplaceable advantages, such as easy processing, light weight, and excellent mechanical properties [1,2,3,4,5,6,7,8]. The rapid development of those modern devices with high power density, high integration, and high voltage has caused escalating hot-spot temperatures, causing a great challenge to the heat resistance of polymer dielectrics present in applications including high-frequency motors, high-voltage transformers, electric vehicles, 5G equipment, and pulsed power apparatuses, etc. [9,10,11,12,13]. However, most of traditional polymer dielectrics are limited to unsatisfactory temperature stability, which usually causes a remarkable deterioration in performance at a high temperature. Therefore, excellent thermal stability, mechanical, and insulating properties become the inevitable requirements for the next generation dielectric materials. Unfortunately, simultaneously integrating these properties portfolios into a single material remains a great challenge.

As one of the high performance fibers, aramid fiber, constructed by highly aligned molecular chains of poly (paraphenylene terephthalamide) (PPTA), is well known for its outstanding mechanical properties, high heat resistance, and excellent electrical insulation properties [14]. At present, aramid fibers and/or aramid pulp fibers are widely used to make insulating papers, but their mechanical properties and dielectric strength are still inadequate due to the poor interfacial interactions between microscale aramid fibers [15,16]. It has been found that aramid fibers can be completely split into uniform high aspect ratio aramid nanofibers (ANFs) by controlled deprotonation [17]. The obtained ANFs inherit the excellent properties of aramid fiber and has emerged as a promising nanoscale building block to fabricate advanced materials owing to its high thermal stability and excellent electrical insulation [17,18,19,20,21,22]. For example, Hu et al. reported a composite film with supreme electromagnetic interference shielding efficiency and exceptional Joule heating performance by combining the ANFs with carbon nanotube and hydrophobic fluorocarbon [23]. Wu et al. and Wang et al. fabricated highly thermoconductive and thermostable polymer nanocomposite films by engineering ANFs with boron nitride nanosheets [1,22]. Zhang and coworkers found that ANFs-based composite films had a potential application as high-performance nanofluidic osmotic power generators [24]. Therefore, incorporating functional fillers into ANFs matrix composite films is an effective strategy to improve its performance. To access the extraordinary properties of ANFs, elegant design the architecture of composites is necessary.

Over the past decade, the aligned “brick-and-mortar” layered structure of nature nacre have demonstrated an effective architecture to achieve remarkable properties. Inspired by the hierarchical microstructures of nature nacre, Zeng et al. successfully fabricated a highly thermally conductive nacre-like papers based on noncovalent functionalized boron nitride nanosheets and poly (vinyl alcohol) via a vacuum-assisted self-assembly technique [25]. Wang et al. claimed that composite films with exceptional insulating properties could be prepared by constructing three-dimensional “brick-and-mortar” layered structures using ANFs and mica nanoplates. As a result, a high dielectric breakdown strength of 164 kV/mm was achieved for the composite film [15]. In addition, the precise design of the inorganic–organic interface is another important factor to fulfill the composite’s properties. Yu et al. proposed a multiscale soft-rigid polymer dual-network interfacial design strategy to reinforce the nanoscale building blocks, which endows the resultant nacreous nanocomposite with superior mechanical enhancement and improved stability under high humidity and temperature conditions [26].

In this study, by learning from the hierarchical microstructure of natural nacre, we fabricated mechanically strong and electrical insulating films by combining ANFs with alumina (Al_2_O_3_) nanoplates using vacuum-assisted filtration, followed by a hot-pressing technique. The underlying rationale for using Al_2_O_3_ nanoplatelets is that Al_2_O_3_ platelets have an excellent dielectric properties with wide band-gap. In addition, the two-dimensional structure of Al_2_O_3_ nanoplates is beneficial for forming a highly ordered arrangement in ANFs framework. Meanwhile, chitosan, a natural cationic polymer obtained by deacetylation of chitin extracted from the shells of shrimp and crabs, has been utilized to enhance the interfacial interaction between ANFs and Al_2_O_3_ nanoplates by constructing hierarchical hydrogen bonds. The ANF/chitosan/Al_2_O_3_ composite film with unique “brick-and-mortar” structure and three-dimensional hydrogen bonds was successfully prepared. The obtained composite film exhibits an ultrahigh electric breakdown strength of 320.1kV/mm and a large elongation at break of 17.22% at 15 wt % filler loading, which was 50.6% and 89.9% higher than those of neat ANFs film, respectively. Moreover, high tensile strength, low dielectric loss, high thermal decomposition temperature are achieved for the composite film simultaneously. It is believed that the biomimetic approach is of great importance for the fabrication and practical application of multifunctional dielectric materials in electrical equipment.

## 2. Materials and Methods

### 2.1. Materials

Al_2_O_3_ nanoplates were purchased from Jicang Nano Technology Co., Ltd., Nanjing, China. Potassium hydroxide (KOH) and carboxylated chitosan were purchased from Aladdin Biochemical Technology Co., Ltd., Shanghai, China. Kevlar^®^ 29 fibers were purchased from DuPont (Wilmington, DE, USA). Dimethyl sulfoxide (DMSO), ethylalcohol, and deionized water (DI H_2_O) were obtained from Chengdu Kelong Chemical Reagent Co., Ltd., Chengdu, China, and were used as received.

### 2.2. Preparation of ANFs, ANF/Chitosan, and ANF/Chitosan/Al_2_O_3_ Composite Films

ANFs were fabricated by treating chopped Kevlar^®^ 29 fibers with a DMSO/KOH solution according to the typical method explored by Kotov et al. [17]. First, 1.6 g of chopped Kevlar^®^ yarn and 2.4 g of KOH were added into the 320 mL of DMSO. Then, the mixture was magnetically stirred at 30 °C at 800 rpm for 1 week, yielding a clear dark red ANF/DMSO dispersion. Then, 100 mL of the obtained ANF/DMSO dispersion was injected into 500 mL of H_2_O to form the colloidal ANF. The filtrate was filtered out with a Buchner funnel, and then the ANF was repeatedly washed with DI H_2_O until the filtrate was neutral, and the purified colloidal ANF was obtained. A stable ANF slurry was obtained by adding 400 mL H_2_O and stirring it at 8000 rpm for 10 min. The pure ANF film was prepared by simple vacuum-assisted filtration with a 0.2 µm pore PTFE membrane. Then, the obtained ANF film was hot-pressed at 150 °C for 5 min and vacuum-dried at 45 °C for 48 h.

The ANF/chitosan composite films were fabricated using the same procedure as for ANFs, with the addition of a certain amount of carboxylated chitosan. Typically, 3 g of carboxylated chitosan was dispersed in DI H_2_O and magnetically stirred for 15 min to obtain a chitosan/H_2_O solution with a concentration of 3 mg/mL, after which other concentrations required could be obtained by dilution with DI H_2_O. The required content of carboxylated chitosan/H_2_O solution was uniformly dispersed in ANF/DMSO solution by sonicating for 3 h, and 500 mL H_2_O was added to obtain ANF/chitosan suspension. The obtained suspension was repeatedly washed with DI H_2_O to make the filtrate neutral. The filtrate was then treated in a high-speed homogenizer at 10,000 rpm for 10 min to obtain homogeneous ANF/chitosan slurry. Then, with the aid of vacuum, ANF/chitosan film was formed on a 0.2 µm pore PTFE membrane. Finally, ANF/chitosan film was further hot-pressed at 150 °C for 5 min and dried at 45 °C for 48 h.

**Figure 1:**
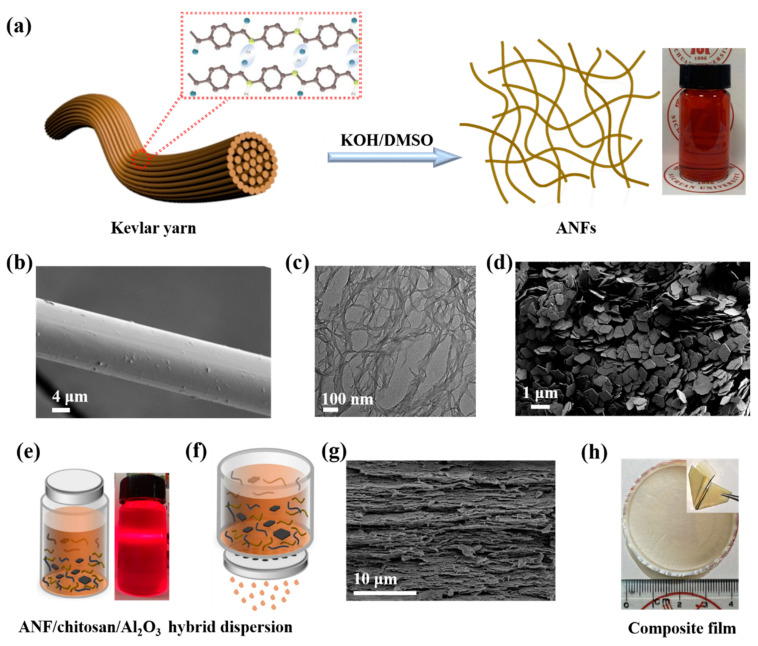
Fabrication of ANF/chitosan/Al_2_O_3_ composite film. (**a**) Schematic diagram for the preparation of ANF using KOH/DMSO dissociating method, and the photograph of the obtained ANF/DMSO dispersion. (**b**) SEM image of Kevlar fiber. (**c**) TEM image of the ANFs. (**d**) SEM image of Al_2_O_3_ nanoplates. (**e**) ANF/chitosan/Al_2_O_3_ hybrid dispersion with strong Tyndall effect. (**f**) Fabrication of composite films by vacuum-assisted filtration. (**g**) SEM image of the cross-section morphology of the ANF/chitosan/Al_2_O_3_ composite film. (**h**) Photographs of ANF/chitosan/Al_2_O_3_ composite films and its mechanical flexibility.

The ANF/chitosan/Al_2_O_3_ composite films were fabricated by a simple vacuum-assisted filtration of a uniformly distributed suspension containing ANFs, chitosan, and Al_2_O_3_ nanoplates. First, The Al_2_O_3_ powder was dispersed in DI H_2_O and then added into the ANF/chitosan suspension. The ANF/chitosan/Al_2_O_3_ slurry was then treated in a high-speed homogenizer at 10,000 rpm for 10 min to obtain homogeneous ANF/chitosan/Al_2_O_3_ slurry. The ANF/chitosan/Al_2_O_3_ films were prepared by direct filtration of the ANF/chitosan/Al_2_O_3_ slurry with the same procedure as ANF/chitosan. The obtained ANF/chitosan/Al_2_O_3_ films were further hot-pressed at 150 °C for 5 min and dried at 45 °C for 48 h. The preparation processes for ANFs and their composite films are illustrated in Figure 1a–h.

### 2.3. Characterization 

The microstructure and morphology of ANF, Al_2_O_3_ nanoplates, and ANF/chitosan/Al_2_O_3_ composite films were characterized by transmission electron microscopy (TEM, JEM2100F, JEOL, Beijing, China) and scanning electron microscopy (SEM, Quanta 250 FEG, FEI, Shanghai, China). Thermal gravimetric analysis (TGA) was performed on the composite films with TG 2950 (NETZSCH, Selb, Germany) at a heating rate of 10 °C/min and N_2_ flow rate of 20 mL/min. The mechanical properties of the composite films were tested at room temperature by the universal testing machine (Instron 5967, Norwood, MA, USA). DDJ-50 kV electric breakdown tester (Kelang Measuring Instrument Co., Ltd., Beijing, China) was used to test the electrical breakdown performance of the composite films at DC high voltage, and the voltage boost rate was 500 V/s. The dielectric response of the composite films was analyzed using the Concept 80 broadband dielectric impedance relaxation spectrometer (Novocontrol GmbH, Montabaur, Germany) in the frequency range of 10^2^~10^6^ Hz. The Nicolet-5700 Fourier transform infrared spectrometer (Thermo Nicolet Corporation, Madison, SD, USA) was used to collect the Fourier transform infrared (FT-IR) spectra of the composite films.

## 3. Results and Discussion

The Kevlar fiber was spilt into ANFs by consistent stirring in a KOH/DMSO system for a week, resulting in a dark red colloidal dispersion as schematically shown in Figure 1a. During the dissociating process, the intermolecular hydrogen-bonding interactions between PPTA molecular backbones were weakened due to the deprotonated effect (Figure 1a, inset) [17,18,27]. Consequently, the original Kevlar fiber with a diameter of ~15 μm (Figure 1b) was dissociating into curly nanofibers with length in micrometer scale and diameter in the range of 20–30 nm, as revealed in the TEM image in Figure 1c. Kotov et al. found that the ANFs not only inherit the exceptional properties of Kevlar fiber, but also possess a large number of functional groups on their surface [17]. The nanoscale, high aspect ratio, surface activity, and good dispersibility of ANFs render them promising nanoscale building blocks to prepare advanced materials [1,22,23,28]. The Al_2_O_3_ nanoplates, with a lateral size of approximately 1 μm and mean thickness of 100 nm (Figure 1d), were utilized to enhance the performance of ANFs based films, owing to their excellent dielectric properties [29,30]. As can be seen from Figure 1e, the hybrid suspension of ANFs, chitosan, and Al_2_O_3_ nanoplates show strong Tyndall effect, which indicates the homogeneous suspension and good interaction between ANFs, chitosan, and Al_2_O_3_ nanoplates. The ANFs/chitosan/Al_2_O_3_ can be made ready by the vacuum-assisted filtration method, as schematically shown in Figure 1f, to form a yellow film with typical lamellar microstructure (Figure 1g), which exhibit excellent flexibility and fold-ability (Figure 1h).

**Figure 2:**
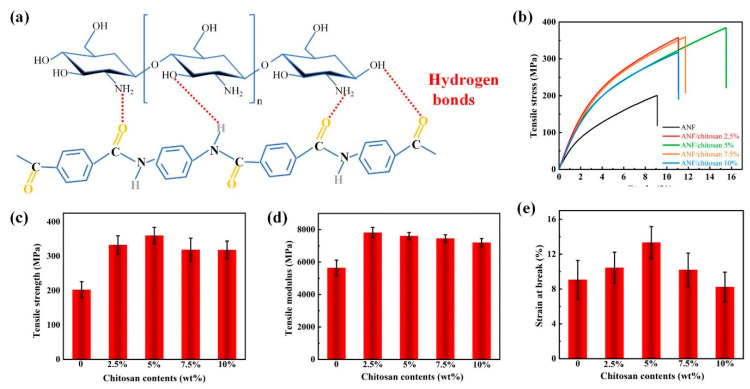
Mechanical properties of ANF/chitosan composite films. (**a**) Schematic representation of the formation of intermolecular hydrogen bonding between chitosan and ANF. (**b**) Typical stress–strain curves of ANF/chitosan composite films at different chitosan contents. (**c**–**e**) Tensile strength, tensile modulus, and break elongation of ANF/chitosan composite at different chitosan contents.

Constructing hydrogen-bonding is a feasible and effective approach to improving the properties of composites [31]. Here, carboxylated chitosan was chosen as molecular modifier to improve the mechanical properties of ANFs, because strong hydrogen bonding can be generated between ANFs and the abundant functional groups (carboxyl, hydroxyl and amino groups) of carboxylated chitosan (Figure 2a). In addition, the carboxylated chitosan not only acts as a hydrogen bonding donor, but also as an interlocking agent to connect the ANFs. Figure 2b,e presents the difference in the mechanical properties of ANF/chitosan composite films with various chitosan contents. It is noted that the mechanical properties of ANF films can be improved remarkably by employing the carboxylated chitosan (Figure 2b). The tensile strength and the elongation at break of the ANF films reach a high value of 360 MPa and 13.34%, which are 78.2% and 47.1% higher than that of neat ANF film (202 MPa and 9.07%), respectively. The intermolecular hydrogen bonding between ANFs and chitosan can be invoked as being responsible for the excellent mechanical properties of ANF/chitosan films. These hydrogen bonds greatly enhanced the intermolecular forces. In addition, due to the existence of hydrogen bonds, the compatibility between ANF and chitosan was brilliant. Chitosan can be uniformly dispersed in ANF, making the internal structure of the composite films compact. The defects and pores are significantly decreased in the compact structure, which is beneficial for enhancing the mechanical properties. The tensile strength and elongation at break of ANF/chitosan composite films decreased slightly when the content of chitosan was higher than 5 wt %, but all of them were higher than that of the neat ANF film. When the content of chitosan was 10 wt %, the tensile modulus of ANF/chitosan composite films decreased by 5.4% (from 7608 MPa to 7201 MPa). The mechanical properties of ANF/chitosan composite films are mainly determined by hydrogen bonding and physical entanglement between soft chitosan molecular chains and relatively rigid ANFs framework. The deterioration of mechanical properties at high chitosan content can be attributed to the saturation of hydrogen bonds and the soft nature of chitosan. Similar phenomena were found in the ANFs/polyvinyl alcohol (PVA) and resol/PVA systems, as reported in the works of E et al. [32] and Chen et al. [26]. Based on the above analysis, when the content of chitosan was 5 wt %, ANF/chitosan composite films showed the best mechanical properties. Therefore, in the present work, we fixed the mass fraction of chitosan as 5 wt % and added more Al_2_O_3_ nanoplates to prepare ANF/chitosan/Al_2_O_3_ composite films.

**Figure 3:**
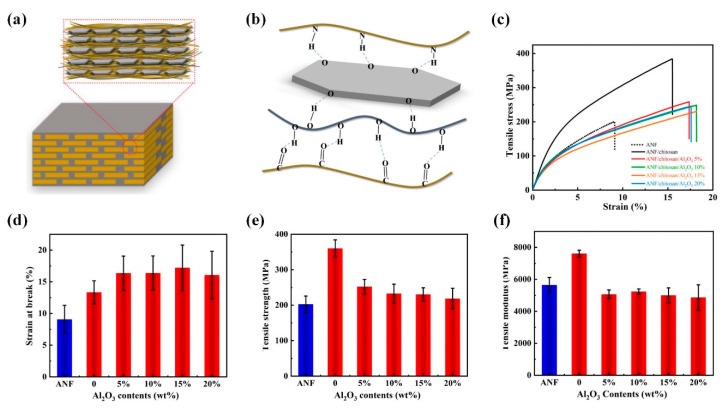
Mechanical properties of ANF/chitosan/Al_2_O_3_ composite films. (**a**) Typical stress–strain curves of ANF/chitosan/Al_2_O_3_ composite films at different Al_2_O_3_ contents. (**b**–**d**) Tensile strength, tensile modulus, and break elongation of ANF/chitosan composite at different Al_2_O_3_ contents. (**e**) Schematic diagrams of the structure of ANF/chitosan/Al_2_O_3_ films. (**f**) Schematic representation of the formation of intermolecular hydrogen bonding between chitosan, ANF, and Al_2_O_3_ nanoplates.

**Figure 4:**
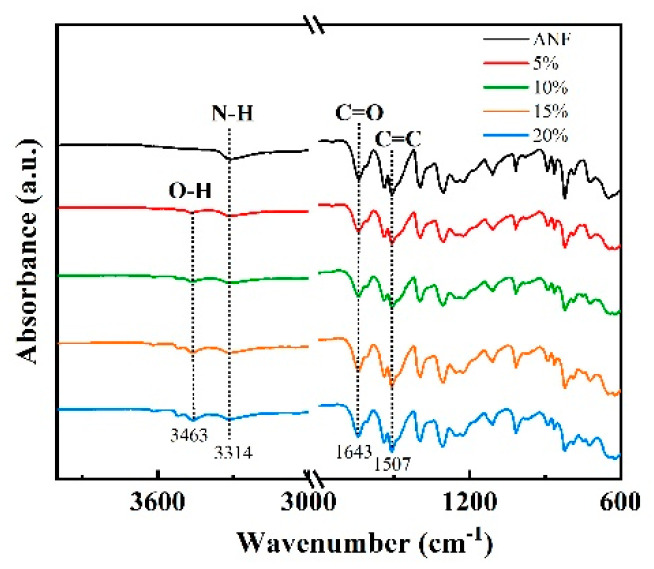
FT-IR curves of ANF/chitosan/Al_2_O_3_ composite films with different contents of Al_2_O_3_ nanoplates.

In the ANF/chitosan/Al_2_O_3_ composite films, the Al_2_O_3_ nanoplates orderly embedded into the framework of the ANFs, and generated a natural nacre-like “brick-and-mortar” structure (Figure 3a). In this special structure, the embedded Al_2_O_3_ nanoplates were glued with ANFs framework together by soft chitosan molecules, and three-dimensional hydrogen bonds were established between ANFs, chitosan, and Al_2_O_3_ nanoplates (Figure 3b). This can be confirmed by means of FT-IR spectra (Figure 4), since the vibrational peaks of functional groups are closely related to the intermolecular interactions. A comparison of the FT-IR spectra of ANF and ANF/chitosan/Al_2_O_3_ shows a remarkable red shift that is representative of the deformation of N–H (from 3316 cm^−1^ of ANF to 3313 cm^−1^ of ANF/chitosan/Al_2_O_3_). This shift is indicative of the interaction between the Al_2_O_3_ and amino (N–H) groups of ANF, resulting in the formation of hydrogen bonds between the ANF and Al_2_O_3_ nanoplates. The nacre-inspired “brick-and-mortar” structure endowed ANF/chitosan/Al_2_O_3_ composites with excellent ductile deformation behavior. As shown in Figure 3c, a large elongation at break of 17.22% was achieved for the ANF/chitosan/Al_2_O_3_ composite film at the filler contents of 15 wt %, which is 89.9% higher than that of neat ANF film (9.07%). The elongation at break was far superior to that of the conventional commercial Nomex insulating paper [15]. As demonstrated in Figure 1g, the composite film could be arbitrarily folded without breakage. The large ductility indicates that the composite film has wonderful manipulation reliability, which is essential to the dielectric materials.

It is noted that the dramatic improvement of the elongation at break of the composite films with the addition of Al_2_O_3_ content is accompanied by a significant decrease in the tensile strength and modulus. Typical stress–strain curves, tensile strength, tensile modulus, and elongation at break of ANF/chitosan/Al_2_O_3_ composite films were shown in Figure 3c–f. The addition of Al_2_O_3_ nanoplates caused a decrease in the tensile strength and modulus of the composite films. The deterioration of the tensile strength and modulus of ANF/chitosan/Al_2_O_3_ can be attributed to the decrease in intermolecular interaction in the composite films. On the one hand, part of the hydrogen bonds between ANF and chitosan were replaced by the hydrogen bonds between ANF/Al_2_O_3_ and chitosan/Al_2_O_3_. Although the new three-dimensional hydrogen bonds were formed, their strength was far lower than that of the hydrogen bonds between ANFs and chitosan. As a result, the intermolecular force was greatly reduced, leading to a decrease in tensile strength of composite films. On the other hand, the free volume of molecular chains is inversely proportional to the compact of the films. The incorporation of Al_2_O_3_ nanoplates will increase the free volume of the composite film, resulting in a decrease in tensile strength. Moreover, the agglomeration will occur with the increase in Al_2_O_3_ nanoplates, which will act as stress concentration point and lead to the degeneration of the mechanical properties. Although the addition of Al_2_O_3_ nanoplates led to the deterioration of mechanical strength, the tensile strength of ANF/chitosan/Al_2_O_3_ composite film at 15 wt % filler contents (232 MPa) was still 14.9% higher than that of neat ANFs film (202 MPa).

**Figure 5:**
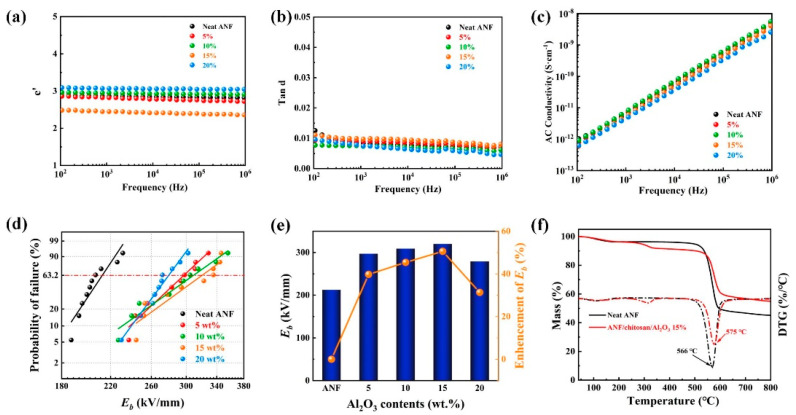
Dielectric properties of ANF/chitosan/Al_2_O_3_ composite films. (**a**) The dielectric constant ($\varepsilon^{\prime }$) and (**b**) loss tangent of ANF/chitosan/Al_2_O_3_ composite films as a function of frequency. (**c**) AC conductivity of ANF/chitosan/Al_2_O_3_ composite films as a function of frequency. (**d**) Breakdown strength of ANF/chitosan/Al_2_O_3_ composite with Weibull distributions. (**e**) The enhancement of the breakdown strength of ANF/chitosan/Al_2_O_3_ composites compared with that of the neat ANF film. (**f**) TGA curves of ANF and ANF/chitosan/Al_2_O_3_ composite films at 15 wt % filler loading.

Next, we turned to investigate the dielectric properties of the ANF/chitosan/Al_2_O_3_ composite films. The frequency-dependent dielectric constants, dielectric losses, and AC conductivity of the composite films are shown in Figure 5a–c. It is observed that the addition of Al_2_O_3_ nanoplates caused a slight increase in dielectric constants of composite film at low filler contents, which can be attributed to the increase in interfacial polarization in ANF/chitosan/Al_2_O_3_ composite films [33,34,35]. In addition, the differential dielectric constants of ANFs and Al_2_O_3_ nanoplates might generate a lot of mini-capacitors, contributing to an increase in the dielectric constant [36]. However, the values of tanδ of the ANF/chitosan/Al_2_O_3_ composite films were below 0.02 (100 Hz, Figure 5b). The AC conductivity of ANF/chitosan/Al_2_O_3_ composites increase linearly with the increase in frequency and no DC plateau is situated in the low frequency (Figure 5c). These results indicate that ANF/chitosan/Al_2_O_3_ composite films possess high insulation capability and low charge carriers mobility [37]. 

The two-parameter Weibull cumulative probability function was utilized to analyze the dielectric breakdown strength of ANF/chitosan/Al_2_O_3_ composite films according to Equation (1):

(1) $$P_{f}=1-\exp\left[\;-\;\left(E/E_{0}\right)\beta \right]$$

where P_f_ represents the cumulative breakdown probability of the electrical system; E is the experimental breakdown strength; β is the shape parameter, reflecting the breakdown voltage dispersion degree; and E_0_ is the characteristic breakdown intensity, reflecting the size of the breakdown field intensity when the cumulative breakdown probability is 63.2%. The dielectric breakdown strength of neat ANF film and ANF/chitosan/Al_2_O_3_ composite films were shown in Table 1 and Figure 5d. It can be observed that the breakdown strength composite films increase remarkably with the addition of Al_2_O_3_ nanoplates. The ANF/chitosan/Al_2_O_3_ composite film with 15 wt % filler contents exhibits the highest dielectric strength of 320.1 kV/mm, which is 50.6% higher than that of neat ANF film (Figure 5e). This indicates that Al_2_O_3_ nanoplates, as a typical dielectrics, can significantly increase the dielectric strength of the ANF film. Similar results are reported in previous research; Zeng et al. have showed that the nacre-mimetics ANF/mica films possess much better dielectric performance than the neat ANF [15]. However, the dielectric strength of ANF/mica is significantly lower than that of ANF/chitosan/Al_2_O_3_ film. This can be attributed to a denser structure of ANF/chitosan/Al_2_O_3_, owing to the hot press under high temperature and high pressure. The prominent improvement of dielectric breakdown strength of composite films was mainly due to the formation of deep traps caused by the addition of Al_2_O_3_ nanoplates. The existence of deep traps can inhibit charge injection and hot electron formation, which will be beneficial for enhancing the dielectric breakdown strength. With the increase in the loading of Al_2_O_3_ nanoplates, the density of the trap increased, leading to the increase in the dielectric breakdown strength. Additionally, the electrons are easier to attract with the wide band gap Al_2_O_3_ nanoplates, leading to more internal charge consumption and less accumulation of space charge in the composite films. As a result, the electric branches migrated to the direction of nanoparticles, which was conducive to the improvement of the breakdown characteristics of the composite films. In addition, the special biomimetic nacreous “brick-and-mortar” structure of ANF/chitosan/Al_2_O_3_ can distribute the electrical stress homogeneously and avoiding the concentration of electrical field. In such a well-arranged architecture, the Al_2_O_3_ nanoplates were orderly embedded into the ANFs framework, which efficiently impedes the growth of electric tree in composite films. Therefore, the breakdown strength of ANF/chitosan/Al_2_O_3_ composite films is significantly improved. However, a decrease in electric breakdown strength is observed when the contents of Al_2_O_3_ are higher than 20 wt %. This can be ascribed to the agglomeration of fillers at high concentration, which will act as a weak point under the high electric field and contribute to the deterioration of the breakdown performance of the composite films. Although there is a slight decrease in electric breakdown strength at high filler loading, the value is still much higher than that of the neat ANF film. Therefore, the ANF/chitosan/Al_2_O_3_ composite films have a great promising as insulating materials application in high-voltage electric power systems. More importantly, ANF/chitosan/Al_2_O_3_ composite film exhibited outstanding thermal stabilities. owing to the high thermal durability of the ANF and Al_2_O_3_ (Figure 5f). Compared to the decomposition temperature (T_d_) of neat ANF film, the T_d_ of ANF/chitosan/Al_2_O_3_ composite film increases by 9 °C from 566 °C of ANF to 575 °C of ANF/chitosan/Al_2_O_3_, which can be attributed to the “tortuous path effect” caused by the “brick-and-mortar” structure. In addition, in the composite film, the Al_2_O_3_ nanoplates preferably absorbed heat due to its high intrinsic heat capacity, which effectively retarded the volatilization of the PPTA chains [22]. 

## 4. Conclusions

In summary, a series of ductile composite films consisting of ANFs, chitosan, and Al_2_O_3_ nanoplates was successfully fabricated by vacuum-assisted filtration followed by hot-pressing. A special biomimetic nacreous “brick-and-mortar” structure was constructed in the ANF/chitosan/Al_2_O_3_ composite films, which effectively restrained the accumulation of space charge and prorogation paths of electric branches in the films. This contributed to a prominent improvement of dielectric breakdown strength of ANF/chitosan/Al_2_O_3_ composite films. An ultrahigh electric breakdown strength of 320.1 kV/mm was achieved for ANF/chitosan/Al_2_O_3_ composite film with 15 wt % Al_2_O_3_ loading, which is 50.6% higher than that of the neat ANF film. In addition, favorable three-dimensional hydrogen bonds have formed between the ANFs, chitosan, and Al_2_O_3_ nanoplates, which imparts an excellent flexibility of composite film, and a large elongation at break of 17.22% was achieved. Furthermore, low dielectric constant, low dielectric loss (<0.02), high tensile strength (~230 MPa), and remarkable thermal stability (Td ~575 °C) were simultaneously achieved for the ANF/chitosan/Al_2_O_3_ composite film. Those admirable features confirmed that the ANF/chitosan/Al_2_O_3_ film, as a typical dielectric material, shows great potential for application in high power apparatuses operating at high temperatures.

## Figures and Tables

**Table 1:** Mechanical properties of ANF and ANF/chitosan/Al_2_O_3_ composite films.

Content (wt %)	*β*	*E_b_* (kV/mm)
0	16.3	212.6
5	10.2	297.1
10	7.7	309.1
15	7.9	320.1
20	14.7	279.2

## Data Availability

The data presented in this study are available upon request from the corresponding author.
